# Cholestatic jaundice in infancy: struggling with many old and new phenotypes

**DOI:** 10.1186/s13052-019-0679-x

**Published:** 2019-07-17

**Authors:** Claudia Mandato, Giada Zollo, Pietro Vajro

**Affiliations:** 1 Department of Pediatrics, Children’s Hospital Santobono-Pausilipon, 80129 Naples, Italy; 2 Pediatrics Section, University of Salerno –Baronissi Campus - Department of Medicine, Surgery and Dentistry, Scuola Medica Salernitana, 84081 Baronissi, Salerno Italy

**Keywords:** Alagille syndrome, ARC syndrome, Cholestasis, Hepatocyte nuclear factor-1-beta, Neonatal cholestasis, Paucity of intralobular bile ducts, Renal cysts and diabetes syndrome

## Abstract

**Background:**

Clinical diagnosis of neonatal cholestasis is considered to be an extremely challenging process. Here we highlight the importance not only of the prompt distinction between extrahepatic and intrahepatic cholestasis forms, but also of the precise identification of the latter ones amongst the hotchpotch of recently discovered metabolic/genetic causes.

Biliary atresia is considered a surgical emergency in a newborn infant. The rate of success in establishing the bile drainage is in fact a function of the early age when the hepato-portoenterostomy intervention is performed. Intrahepatic cholestasis is due to a broad and more and more puzzling variety of infectious, endocrine, genetic, metabolic and toxic disorders where Gamma-glutamyl transpeptidase serum levels may help for differential diagnosis. Recently established laboratory diagnostic techniques have allowed to discover new causes of neonatal cholestasis. Aim of the Commentary is to go through some of them and bring the focus particularly on the information deriving from the paper by Pinon et al. in this issue of the Journal, which paves the way to the inclusion of the hepatocyte nuclear factor-1-beta deficiency as a new condition to consider in the diagnostic process of the syndromic forms with paucity of intralobular bile ducts.

**Conclusion:**

Neonatal cholestasis poses diagnostic challenges in practice. Recent advances in the pathophysiology and in molecular genetics together with clinical features, histopathologic findings and careful reasoning remains paramount to put together the pieces of the jigsaw.

## Background

**Fig. 1:**
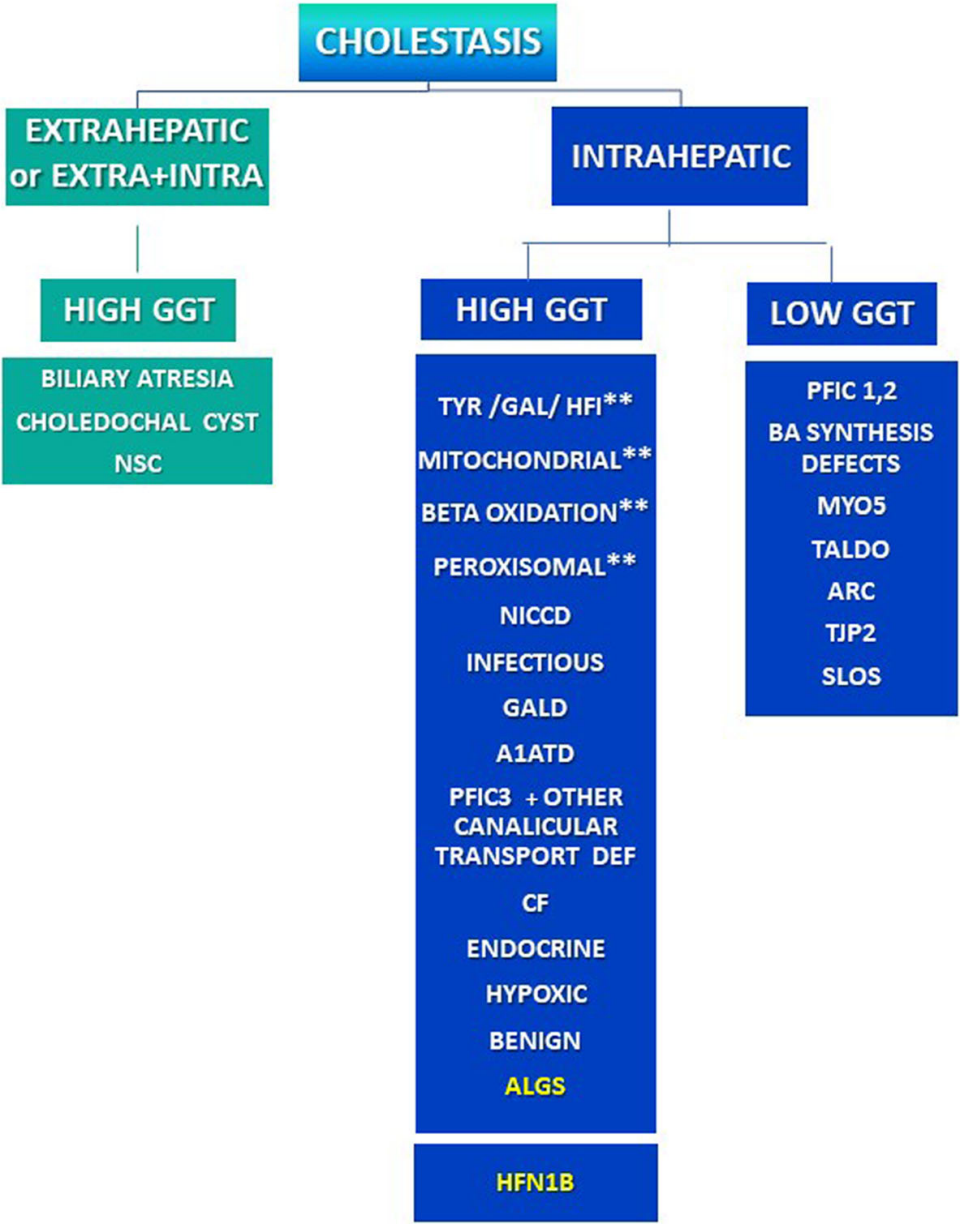
Schematization of the causes of neonatal cholestasis. Patients with Renal Cysts and Diabetes Syndrome (RCAD) may present a syndromic paucity of interlobular bile ducts with high GGT neonatal cholestasis possibly mimicking Alagille Syndrome (ALGS). *A1ATD* Alfa-1 Antitrypsin Deficiency, *ALGS* Alagille Syndrome, *ARC* Arthrogryposis, Renal Dysfunction and Cholestasis, B*MYO5* Myosin V, CF Cystic Fibrosis, *GAL* Galactosemia, *GALD* Gestational Autoimmune Liver Disease (Neonatal Hemochromatosis), *GGT* Gamma-glutamyl transpeptidase*, HFI* Hereditary Fructose Intolerance, *HFN1* hepatocyte nuclear factor 1 homeobox, *NICCD* Neonatal Intrahepatic Cholestasis Caused by Citrin Deficiency, *NSC* Neonatal Sclerosing Cholangitis, *PFIC* Progressive Familial Intrahepatic Cholestasis, *RCAD* Renal Cysts And Diabetes Syndrome, *SLOS* Smith-Lemli-Opitz Syndrome, *TALDO* Transaldolase Deficiency, *TYR* Tyrosinemia, *TJP2* Tight Junction Protein2. ** TYR, GAL, HFI as well as GALD and some mitochondrial, peroxisomal and beta oxidation diseases may have a liver failure presentation

Neonatal cholestasis affects approximately 1 in every 2500 term infants. It is defined as a reduced bile formation or flow leading to the retention of biliary substances which should instead be excreted into bile and eliminated through the intestinal lumen [1, 2]. Typical laboratory features are commonly represented by cholalemia and conjugated hyperbilirubinemia. The latter is defined as a conjugated bilirubin serum level > 1 mg/dL [3]. On the clinical side, in addition to jaundice, patients present some relevant red flags such as hyperchromic urines, hypocholic or acholic stools, and sometimes incipient itching. Neonatal cholestasis may stem from several conditions, such as obstructive anatomic abnormalities of bile ducts, infections, genetic and metabolic diseases, sepsis, endocrinopathies, perinatal asphyxia or parenteral nutrition, the latter ones especially in preterm infants. (Figure 1) Some forms may remain of undetermined origin (so called, with descriptive term, “idiopathic neonatal hepatitis”).

Due to the broadness of this puzzling scenario, reaching a rapid aetiological diagnosis may represent an extraordinary clinical challenge. In any case, making an early diagnosis remains extremely important to determine a better outcome, and, therefore,the American Academy of Pediatrics and the North American and European Associations for Pediatric Gastroenterology, Hepatology and Nutrition recommend that infants with jaundice longer than 2–3 weeks should have tested both conjugated and total bilirubin, and be evaluated by an expert as soon as possible [3, 4].

## Clinical diagnostic evaluation

**Fig. 2:**
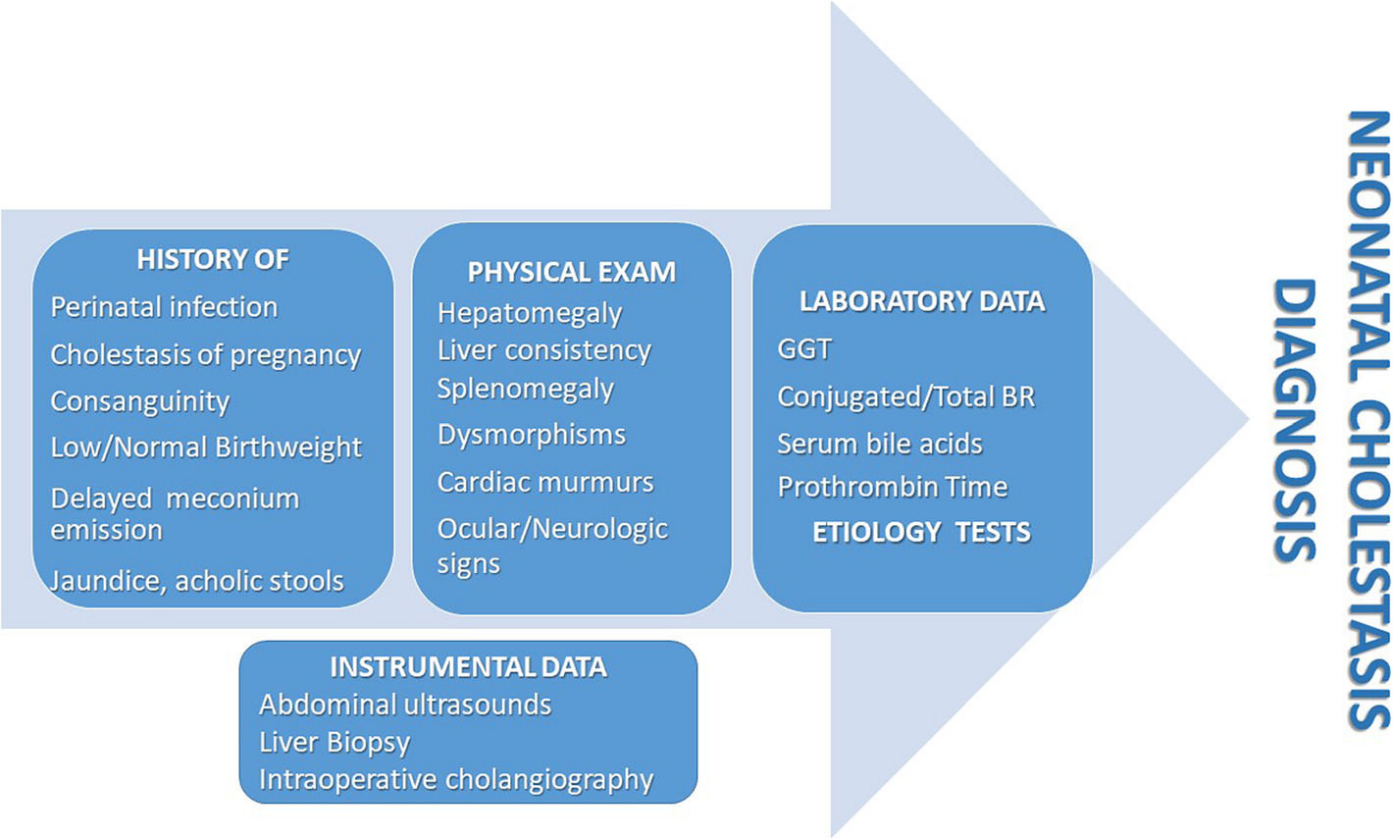
Anamnestic, clinical, laboratory and instrumental data which may help in addressing the diagnosis of neonatal cholestasis. *BR* Bilirubin, *GGT* Gamma-glutamyl transpeptidase

Diagnosis derives from anamnestic and clinical elements, as well as laboratory and instrumental data **(**Fig. 2**)**. Relevant anamnestic elements should include history of maternal infection or cholestasis during pregnancy, which may be due to the carriage of Progressive Familial Intrahepatic Cholestasis (PFIC) genes. Delayed meconium emission may indicate cystic fibrosis, while a history of consanguinity may suggest a genetic/inherited metabolic disease. Timing of jaundice onset and changes in stool and urine pigmentation may offer clues to the differential diagnosis. Clinical elements to be sought after are the presence of hepatomegaly and liver hardness which may result increased in extrahepatic cholestasis. Splenomegaly may indicate storage disorders (e.g. Niemann-Pick type C disease), while cardiac murmurs and/or renal involvement may suggest either Tyrosinemia or Alagille Syndrome (ALGS). Neurologic signs and symptoms may bring into play some metabolic/mitochondrial disorders, sepsis or acute liver failure. Severe coagulopathy in neonates has been described as the consequence of sepsis or of acute liver failure especially within the historical group of metabolic diseases such as galactosemia, hereditary fructose intolerance and tyrosinemia [2].

## Laboratory evaluation

Laboratory evaluation typically detects an aspecific increase of conjugated and total bilirubin levels, often associated to significant elevation of Gamma-glutamyl transpeptidase (GGT) and cholalemia. It must be remarked however that GGT levels can be normal or low in PFIC type 1 and 2 and in a number of other rare cholestatic conditions **(**Fig. 1**)**. Total serum bile acids are usually elevated except in bile acid synthesis defects which also have low GGT values. While hypertransaminasemia indicates hepatic damage, low albumin levels together with prolonged prothrombin time are highly suggestive of liver failure.

## Is it extrahepatic cholestasis?

The archetypal types of exclusive/prevalent extrahepatic cholestasis are biliary atresia (BA) and choledochal cyst **(**Fig. 1**)**. Usually, infants with BA appear healthy and well thriving in spite of persistent jaundice and completely and persistently acholic stools, so that the unremarkable clinical conditions may sometimes result misleading. Up to 20% of them however display associated congenital malformations, including heterotaxy with polysplenia, *situs viscerum inversus*, vena cava agenesis which may help to orientate a correct and more prompt diagnosis. The rate of success in establishing bile drainage in infants with BA is a function of the early age of the infant when the hepato-portoenterostomy intervention (Kasai procedure) is performed, so that BA is considered a surgical emergency in a newborn.

## Is it intrahepatic cholestasis?

Intrahepatic cholestasis is due to a broad and more and more puzzling variety of disorders. GGT serum levels may help in the differential diagnosis. A low or normal GGT can typically be found in PFIC types 1 and 2, or Bile Acids synthesis defects. As shown in Fig. 1, low levels of GGT however are also associated with a number of other rare cholestatic conditions like Smith Lemli Opitz syndrome, Arthrogriposis-Renal dysfunction and Cholestasis (ARC syndrome), Transaldolase Deficiency, and mutations in the Tight junction protein 2 gene (*TJP2*) which cause failure of protein localization and microvillus inclusion disease. A high level of GGT may suggest a hotchpotch of intrahepatic metabolic/genetic diseases such as α1-antitrypsin deficiency, neonatal (extra and intrahepatic) sclerosing cholangitis, ALGS, cystic fibrosis, PFIC type 3 and other canalicular transport defects, as well as endocrinopathies or hypoxic neonatal cholestasis. In subjects who are acutely ill, one should consider tyrosinaemia, galactosaemia, hereditary fructose intolerance as well as GALD [Gestational Autoimmune Liver Disease or Neonatal Hemochromatosis], and some mitochondrial, peroxisomal and beta oxidation diseases. Thanks to the next generation sequencing, genetic tests for most of these metabolic/genetic conditions are now also commercially available (so called liver panel) [5].

## Paucity of intralobular bile ducts and Alagille syndrome

The hepatobiliary lesion in ALGS (OMIM#118450) consists in paucity of intralobular bile ducts (PILBD). It is a rare multi-systemic autosomal dominant disorder, with mutations of the JAG1 (20p12) pleiotropic gene encoding a Notch signaling pathway ligand (ALGS type 1) or (more rarely, in about 1% of patients) of the NOTCH2 gene (1p12) (ALGS type 2) [6–8]. Remarkably, still 5–7% of ALGS patients have a partial or complete deletion of JAG1, which may not be detected by next generation sequencing since it requires more specific test for structural rearrangements, e.g. multiplex ligation-dependent probe amplification [5]. Being a dominant condition, ALGS has an incomplete penetrance, determining a significant phenotypic variability within affected patients that can lead to difficulties in diagnosis, especially in recognizing mildly affected individuals [9]. In addition to PILBD, clinical diagnosis is typically supported in presence of three out of the following five clinical criteria: liver disease or cholestasis; heart defect; skeletal abnormality; ophthalmologic abnormality; distinctive facial features. Although less frequent, two other criteria, namely vascular abnormalities and structural (e.g. small hyperechoic kidney and renal cysts) as well as functional renal abnormalities common in ALGS type 2, have been described and have been proposed to contribute to the correct allocation of patients with ALGS. In this regard an expansion of the phenotypic criteria of ALGS such that three out of seven characteristic clinical criteria have been proposed to be sufficient for a clinical diagnosis [7].

## Paucity of intralobular bile ducts and RCAD syndrome

In this issue of the Italian Journal of Pediatrics, Pinon et al. [10] put the light on another syndromic condition which also appears to be characterized by cholestasis due to a (hitherto almost overlooked in the literature) hepatic picture of PILBD, which is histologically similar to that of ALGS and is combined with renal involvement as well [10]. This association which brings to mind a *fruste* form of ALGS however has not JAG1 and NOTCH2 genes mutations, but has instead pathogenic HNF1β (hepatocyte nuclear factor-1-beta) gene mutations. HNF1β, a homeobox transcription factor, also plays a pivotal role in organogenesis during embryonic stage. This recently recognized syndromic disorder presents a variable multisystem phenotype known as *Renal Cysts and Diabetes Syndrome* (RCAD, OMIM #137920). In spite of the name which is due to the characteristics of early reports, however, it is now evident that diabetes and renal cysts are not always present and instead it is associated to a large phenotypic spectrum including pancreatic, urogenital, neurological, parathyroid involvement and – although less typically- liver involvement as well. The latter is now known to encompass from an asymptomatic hypertransaminasemia to a cholestatic liver impairment, including neonatal or late-onset cholestasis [11].

In this regard, Pinon et al. [10], by accurately reviewing the available literature, brought into focus that five patients previously described with HNF1β deficiency, similarly to their own case, had a histological findingof PILBD at the basis of their cholestatic picture [8]. Their article for the first time puts therefore forward the suggestion that when facing to possible mildly affected syndromic cholestatic individuals with negative genetic study for Jagged 1 and Notch genes, one might be dealing instead with the hepatic component of RCAD. Recent data indicate that HNF1B mutations result responsible also for ∼10% of congenital abnormalities of kidney and urinary tract cases, both in children and in adults [2]. We are presently unaware of their precise contribution to the hepato-renal phenotypes: we need therefore to implement the recognition of this clinical entity which is probably still undiagnosed both by adult and pediatric specialists [12]. This is relevant in that it represents an obvious premise for correct counseling of patients and their families, allowing also prenatal diagnosis, and evaluation of family members as potential donors for a future liver transplantation.

## Conclusions

Clinical diagnosis of pediatric onset cholestasis all in all remains extremely challenging [2]. One must be careful and remain very aware of the possible contribution of newer diagnostic techniques which may allow to individuate HNF1B deficiency as a novel tile in the jigsaw of the differential diagnosis of PILBD based cholestasis.

## Data Availability

Not applicable.

## Abbreviations

ALGS: Alagille Syndrome
ARC: Arthrogriposis-Renal dysfunction
BA: biliary atresia
GGT: Gamma-glutamyl transpeptidase
HNF1B: hepatocyte nuclear factor-1-beta
OMIM: Online Mendelian Inheritance in Man
PFIC: Progressive Familial Intrahepatic Cholestasis
PILBD: Paucity of intralobular bile ducts
RCAD: Renal Cysts and Diabetes Syndrome
TJP2: Tight junction protein 2 gene
